# New synonymy of *Proceratium williamsi* Tiwari (Hymenoptera, Formicidae)

**DOI:** 10.3897/zookeys.388.6972

**Published:** 2014-03-13

**Authors:** Himender Bharti, Aijaz Ahmad Wachkoo

**Affiliations:** 1 Department of Zoology & Environme; 2 ntal Sciences, Punjabi University, Patiala - 147002, India

**Keywords:** Ants, *Proceratium bhutanense*, *Proceratium williamsi*, synonymy, India

## Abstract

*Proceratium bhutanense* De Andrade, 2003, **syn. n.** is here found to be conspecific with *Proceratium williamsi* Tiwari, 2000 and accordingly treated as a junior synonym.

## Introduction

At present 79 extant and 5 fossil species are listed in the ant genus *Proceratium* across the globe (Bolton et al. 2007; Bolton 2012). In India, this genus is represented by two species (Bharti 2011). *Proceratium williamsi* was described by Tiwari (2000) as the first record of the genus from India; shortly afterwards De Andrade (2003) added *Proceratium bhutanense* to the Indian *Proceratium*.

Unfortunately, Baroni Urbani and De Andrade (2003) left out *Proceratium williamsi* from their global taxonomic revision of *Proceratium*, possibly due to lack of access to an obscure paper published locally. However, re-examination of both Indian species finds them conspecific. The specimens of *Proceratium williamsi* collected by R. Mathew from the type locality Meghalaya, Khasi hills, Shillong also form part of the material examined for *Proceratium bhutanense*. Descriptions, morphometrics, line drawings, images and collection localities of two are also akin. Therefore, *Proceratium bhutanense* is considered here as a junior synonym of *Proceratium williamsi*.

## Material and methods

The morphological observation was conducted on a Nikon SMZ 1500 stereo zoom microscope. For digital images, MP evolution digital camera was used on the same microscope with Auto-Montage (Syncroscopy, Division of Synoptics, Ltd.) software. Later, images were cleaned as required with Adobe Photoshop CS5.

Abbreviations of the specimen depositories are:

**BMNH** The Natural History Museum, London, England, U.K.

**MRSN** Museo Regionale di Scienze Naturali, Torino, Italy.

**NHMB** Naturhistorisches Museum Basel, Switzerland.

**PUPAC** Punjabi University Patiala, Ant Collection, Patiala, India.

**ZSIK** Zoological Survey of India, Kolkata, India.

## Results and discussion

### Proceratium williamsi

Tiwari, 2000

http://species-id.net/wiki/Proceratium_williamsi

Figs 1–3

Proceratium williamsi Tiwari, in Mathew and Tiwari 2000: 272, Figs 14–15 (w.). Holotype and paratype workers: Meghalaya, Khasi hills, Shillong, India [ZSIK].
Proceratium bhutanense De Andrade, in Baroni Urbani and De Andrade 2003: 278, Figs 116–117 (w.). Holoype and paratype workers: Phuntsholing, Bhutan [NHMB]; one paratype worker Phuntsholing, Bhutan [MRSN]. Syn. n.

#### Material examined.

Paratype, worker, Meghalaya, Khasi hills, Shillong, India [ZSIK]; Worker [BMNH] (coll. R. Mathew, Det. De Andrade); worker photographs also examined on AntWeb (www.antweb.org): CASENT0281860. *Other Material*: *Uttarakhand*: Dakpathar, 750m, 4 (w.), 20.viii.2009; Rajaji Forest Area, 660m, 3(w.), 11.viii.2009, 1(w.), 12.viii.2009 (coll. Aijaz A. Wachkoo) [PUPAC]. *West Bengal*: Darjeeling, 1850m, 4(w.), 20.vi.2009 (coll. Irfan Gul) [PUPAC]. *Meghalaya*: Cherapunji, 1200m, 3(w.), 2.iv.2009 (coll. Irfan Gul); Khasi hills, Shillong, 1496m, 3(w.), 1(q.), 10.iv.2009 (coll. Irfan Gul) [PUPAC].

**Figures 1–3.**
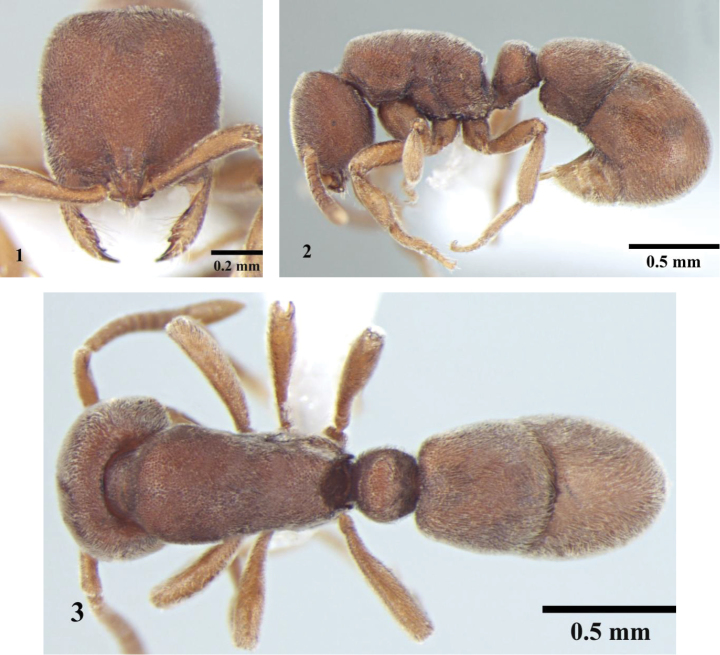
Worker; *Proceratium williamsi* Tiwari, 2000. **1** Head in full-face view **2** Body, lateral view **3** Body, dorsal view.

#### Ecology.

This species was found mainly in leaf litter of primary, subtropical forests of Himalaya and occasionally in soil samples of secondary forests collected in cool shady places. Although infrequent in collections, this species seems to be widely distributed throughout the Himalayan ranges.

#### Remarks.

Examination of the specimens coupled with the images and descriptions reveal that there are no characters which could delimit *Proceratium bhutanense* and *Proceratium williamsi*. Moreover, the studied material does not exhibit any marked variation throughout the collection range, thereby enabling us to confidently treat *Proceratium bhutanense* as a junior synonym of *Proceratium williamsi*.

## Supplementary Material

XML Treatment for Proceratium williamsi
